# A fast secure and more reliable underwater communication system based on light fidelity technology

**DOI:** 10.1038/s41598-025-96484-8

**Published:** 2025-04-23

**Authors:** M. Sami Ataa, Eman E. Sanad

**Affiliations:** Faculty of Computers and Artificial Intelligence (FCAI), Cairo University, Giza, Egypt

**Keywords:** Li-Fi, Laser diodes, VLC, AUVs, CRC, AES

## Abstract

Underwater communication is essential for industries such as marine research, offshore exploration, defense, and telecommunications. Traditional methods, including acoustic and radio waves, suffer from bandwidth limitations, high latency, and interference. Light Fidelity (Li-Fi) technology presents a promising alternative, offering high bandwidth and reduced interference. However, challenges such as signal attenuation, environmental obstacles, and security vulnerabilities must be addressed to ensure reliable and secure communication. This paper proposes a Li-Fi-based underwater communication system that enhances data reliability through deterministic Line-of-Sight transmission and minimal external interference. Security is reinforced using the Advanced Encryption Standard and reliability is ensured through the Cyclic Redundancy Check algorithm. The system successfully transmits various data formats, including text, images, and GIFs. Our findings demonstrate the feasibility of Li-Fi for underwater applications, highlighting its adaptability and potential to overcome existing communication challenges. We aimed to provide a comprehensive understanding of the underlying principles of Li-Fi technology and its adaptability to the challenges posed by underwater communication environments.

## Introduction

The exploration and utilization of underwater environments have gained substantial attention due to their critical roles in scientific research, resource extraction, marine ecology monitoring^1^, and defense operations. As humans venture deeper into the seas and oceans, the demand for efficient and secure underwater communication becomes paramount. Traditional wireless communication methods, primarily reliant on Radio Frequencies (RFs), exhibit severe limitations when deployed underwater. The unique properties of water, such as its high attenuation and absorption of electromagnetic signals, pose complicated challenges to establishing reliable and secure communication links.

The emergence of Li-Fi, a groundbreaking optical wireless communication technology, offers a promising solution to these challenges. The demand for Li-Fi is expected to increase in the near future due to its convenient characteristics, which make it suitable for a wide range of applications^2^.

Li-Fi exploits visible light as the communication medium. It operates on the principle of modulating light intensity at rapid rates, imperceptible to the human eye, to transmit data. This technology enables wireless communication with higher data rates, improved efficiency, greater availability, and enhanced security^3^ compared to radio frequency (RF) communication. Despite these advantages, light-emitting diode (LED)-based technologies have not been widely adopted for wireless communication, as further research is still needed^4^. Unlike radio waves, light signals can propagate through water with minimal attenuation, making Li-Fi an attractive candidate for underwater communication and many other applications such as smart lighting and vehicular communication. This technology addresses the limitations of RF underwater communication. However, challenges remain, such as overcoming range limitations and mitigating interference between different light sources^5^. Also, Li-Fi emerges as a promising solution to the growing depletion of the RF spectrum by utilizing visible light for data transmission^6^.

Both Light waves and Radio waves are types of electromagnetic waves with different wavelengths, frequencies, and other characteristics which lead us to choose Light waves instead of Radio waves^7^. A comparison between the two types of waves shown in Table 1 can highlight some key differences that led to this choice. First, light waves operate at much higher frequencies and shorter wavelengths than radio waves, leading to more precise data transmission with lower power consumption. Second, light waves have lower latency, higher data rates, and a lower cost than radio waves. Moreover, light waves are less affected by Electromagnetic (EM) interference, making them more reliable in environments with high EM noise.

**Table 1:** Comparison between radio waves and light waves^8,9^.

Point of comparison	Radio waves	Light waves
Frequency	3KHZ–300 GHZ	430–790THZ
Wavelength	1 mm–100 m	380–750 nm
Power consumption	Almost 100w	Almost 10w
Latency	Moderate	Low
Data rate	< 0.1 Gbps	< 10 Gbps
Cost	High	Low
Outsource affection	Affected by EM sources	Not affected
Categories	TIF, ELF, LF, MF, EHF, THZ	Infrared, Visible light, UV
Applications	Satellite, TV broadcasting	Lighting, communication

Underwater communication utilizes two main methods light waves, as seen in Li-Fi, and acoustic waves, commonly known as sonar^10^. A comparison between the two technologies shown in Table 2 emphasizes that light waves, used in Li-Fi offer high-frequency, short-wavelength transmission with stable power consumption, lower latency, a significantly higher data rate, lowest cost, and minimal interference. In contrast, sonar, which uses acoustic waves, operates at lower frequencies and longer wavelengths but is more susceptible to environmental fluctuations, such as amplitude variations.

**Table 2:** Comparison between light waves and sonar^9,11^.

Point of comparison	Light waves	Acoustic “sonar”
Frequency	430–790THZ	25–250 KHZ
Wavelength	380–750 nm	16.5 mm–1.65 m
Power consumption	Almost 10w	Almost 10w
Latency	Low	High
Data rate	< 10 Gbps	< 10 Kbps
Cost	Low	High
Outsource affection	Not affected	Affected by Amplitude fluctuation
Categories	Infrared, Visible light, UV	Leaky surface acoustic waves, Rayleigh waves, Electroacoustic waves
Applications	Lighting	Direction guide

Considering the previously highlighted advantages of Li-Fi, the increasing scarcity of the RF spectrum, and the expanding range of applications including underwater communication further research, and development in this field are crucial to overcoming existing challenges, while introducing Li-Fi as a powerful technology.

The remainder of this paper is organized as follows. Section Related work discusses some of the related works in the research scope. Section Methodology describes the methodology used in this research. Section Proposed system development offers the proposed system and the results of the experimental work. Section Proposed system use cases highlights some of the most valuable use cases that can implement the proposed system. Finally, section Conclusion and future work gives the conclusion and the future work.

## Related work

Underwater communication has been a subject of extensive research. Light Fidelity (Li-Fi) technology has emerged as a promising alternative due to its high-speed data transmission and reduced interference. Several studies have explored the use of Li-Fi in underwater environments, each addressing different aspects of communication. This section reviews some of the existing works that tried to build a Li-Fi communication system that can be used for underwater purposes.

The proposed system in this research was motivated by the concepts presented in the FCAI students’ graduation project (2022) at Cairo University, in which an underwater temperature sensor system was built using Li-Fi. The system was so simple that its only function was to sense the temperature of the water then convert the data from hexadecimal to binary and send the 5-bit data through laser.

The system faced issues such as allowing a small size of data and only one format to be sent. It also has a fixed sender, which contains the sensor, and a fixed receiver, which contains a solar panel to collect data. It has no reliability feature, so it’s not known if the data has been received or not. The most important issue is the absence of any security techniques to secure the data in the system.

In^12^ Biswas et al. introduced a system that includes an Arduino Uno chip, a light-dependent resistor, and a light-emitting diode. Their work focuses on leveraging the capabilities of the Arduino Uno chip, without any other considerations.

In^13^ Thilagavathy et al. utilize a booster to strengthen the light so can travel further. They used an Arduino board for data processing, transmission, and reception. They also managed to send and receive text, audio, and video. The authors did not mention any security or reliability mechanisms.

In^14^ Bin Rashid et al. developed a Li-Fi communication system for their underwater vehicle to provide them with real-time data for visualization. The main aspect of the authors was the vehicle, not the communication system.

In^15^ Karthikeyan et al. proposed a diving health monitoring system, which depends on Li-Fi for sending health sensors’ measurements. The authors focused on studying the optical signal propagation problem, not on building a comprehensive system.

In^16^ Y. R. Lata et al. suggest a half-duplex communication system based on Li-Fi technology for short-distance underwater communication. Their system managed to send both text and audio files, they also proposed a remote controller feature for underwater equipment. They tested their system using a swimming pool. However, they did not mention any security features for their system.

In^17^ Thorat et al. made a scenario-based Li-Fi system design with unit coverage area measured as cells. They can control the coverage area by considering cell size, cell shape, and light intensities. They proved that the throughput when implemented on a macro or even a wider area network scale has a higher probability of compensating. Their proposed system wasn’t physically tested so the numbers weren’t so accurate.

In^18^ Krishnamoorthy et al. modeled the underwater channel using the ray tracing method, as described. The authors modulate the data using Binary Phase Shift Keying (BPSK) modulation. The simulation of both transmitter and receiver is carried out using MP-Lab. The hardware implementation is done using a PIC microcontroller to process data. A 50-watt bulb and Olud speaker are used as transmitter and receiver. The whole system was automated using Bluetooth, IoT, and GPS tracker.

In^19^ M. Amen et al. provide a simple yet speedy system based on Li-Fi technology. That can be used for underwater applications. The main target for the authors is to reduce the consumed power during the communication process.

In^20^ Imran et al. tried to send audio, video, text, and image data with a successful rate of 100%. The authors tried different distances and they figured that a high-resolution image can be easily transmitted between a sender and a receiver that has 12 feet distance in between. The authors did not mention any security or reliability techniques.

In^21^ S. Rajan et al. implemented a novel underwater communication system that reaches up to 100 meters distance. Their system was evaluated using different underwater scenarios to come up with the highest-speed communication system. There is no mention of any transmitted data types nor any security or reliability technique

In^22^ M. Abdhulkader et al. conducted a comparison between Wi-Fi and Li-Fi, which found that the data rate is 10Mbps higher in Li-Fi. The authors claim that Li-Fi in its basic form is a more secure technique than Wi-Fi. Also, they used sound as the transmitted data.

In^23^ S. Mahalakshmi et al. built a Li-Fi-based communication system using two Arduino Nano microcontrollers. The authors used sensor data such as heart rate, temperature, and humidity. They utilized a full-duplex system with a real-time alert sensor.

In^24^ J. Mahakal et al. managed to send and receive text, audio, and images below the water. The authors try different environments such as air, clean water, salty water, and muddy water for testing their system. The results show that text data cannot be sent through muddy water and it has 80% accuracy when using salty water.

In^25^ J. Shirisha et al. proposed a Li-Fi communication system for sending both text and audio files. The aim of the study is to understand the different characteristics of the Li-Fi technology. The authors do not mention testing their system using a water environment, nor using any technique for enhancing the security the reliability of the system.

In^26^ R. Seetharaman et al. aimed to develop a communication system for various underwater applications. The system uses Li-Fi as a communication technology, and Arduino as a microcontroller. The authors do not mention anything about the types of data being transmitted, any security techniques, or any reliability techniques.

The reviewed literature highlights various implementations of Li-Fi-based communication systems, each addressing specific aspects such as data transmission efficiency, power consumption, and environmental adaptability, signal boosting, or underwater vehicle communication. Several studies demonstrated the successful transmission of different data types, including text, audio, video, images, and sensor data, in both ground and underwater environments. However, a common limitation among these works is the lack of focus on security protocols and reliability techniques. Also, some researchers did not extend their research to underwater conditions at all. The existing studies either focus on the feasibility of Li-Fi for communication or its performance in specific scenarios but fail to present a comprehensive solution that ensures high-speed, secure, and reliable communication in challenging underwater environments. This research aims to bridge these gaps by enhancing Li-Fi applicability in underwater communication through improved data transmission techniques, reliability techniques, and robust security protocols,

## Methodology

The research follows a structured approach to establish a secure and reliable underwater communication system using Li-Fi technology. The research also aims to try to send different types of data files including text, images, audio, and video.

The methodology of this research is based on a step-by-step technique, where the initial step of the proposed system involves establishing a foundational framework for communication between the sender and receiver, initially limited to text-based data transmission. This step revolves around ensuring reliability by employing echo messages to verify the successful delivery of messages between the two users.

Subsequently, the proposed system advances to the second step, which focuses on enhancing the security of communication through the implementation of basic cryptography techniques such as Affine-cipher. Further improvements in reliability and security are achieved through the incorporation of the Cyclic Redundancy Check (CRC) error detection algorithm, and the Advanced Encrypted Standard (AES) security algorithm.

The proposed system then progresses to more diverse data formats, commencing with the transmission of grayscale encrypted images. Following this, the subsequent step involves extending the communication capabilities to accommodate the transmission of grayscale videos, a small-scale video is used which is a grayscale encrypted Graphics Interchange Format (GIF) file.

## Proposed system development

To address the challenges and limitations identified in previous research, we propose an enhanced Li-Fi-based underwater communication system that prioritizes both reliability and security. The system is designed and tested to facilitate the efficient transmission and reception of different types of data. The proposed system integrates cryptographic techniques and error detection mechanisms to ensure secure and reliable communication. The following sections provide a detailed overview of the system’s architecture, components, and functionality.

**Fig. 1:**
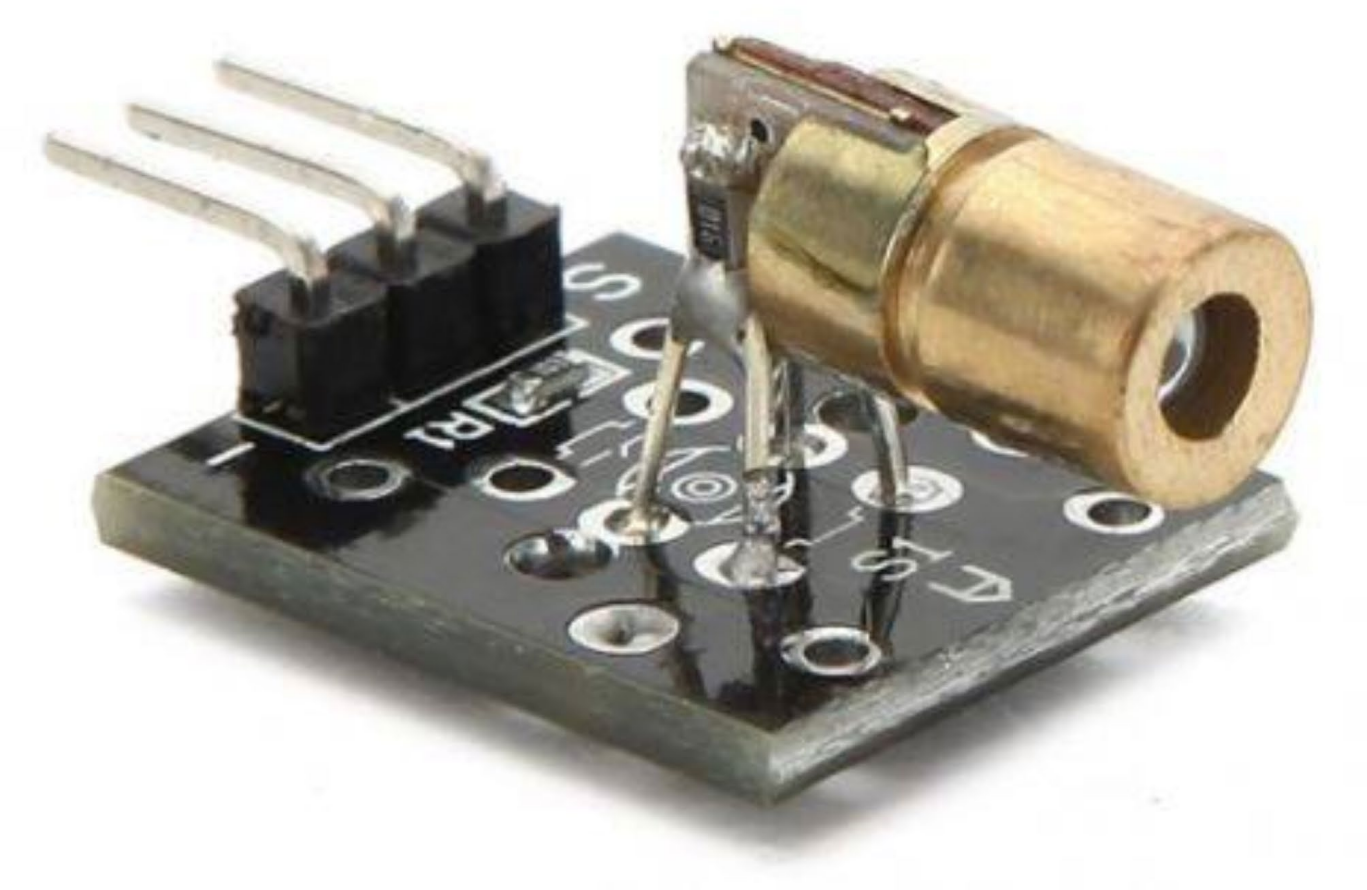
Laser transmitter module^27^.

**Fig. 2:**
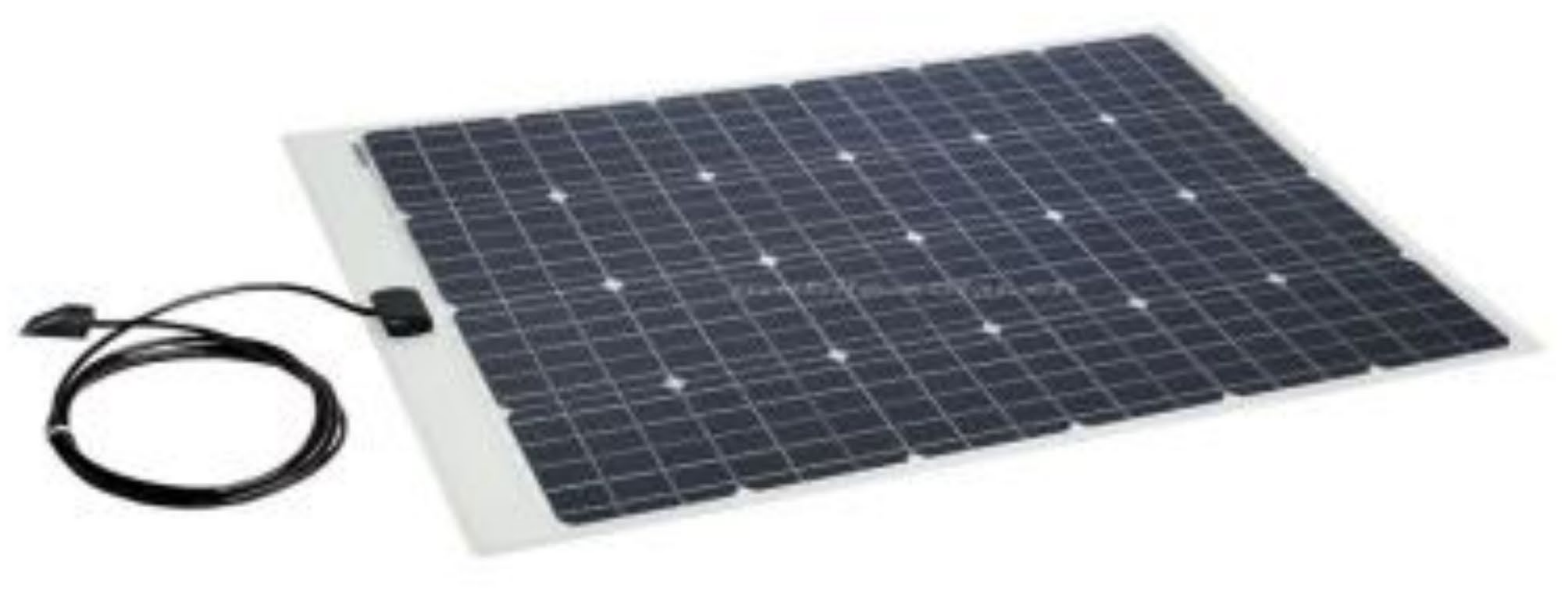
A light Collector panel^28^.

**Fig. 3:**
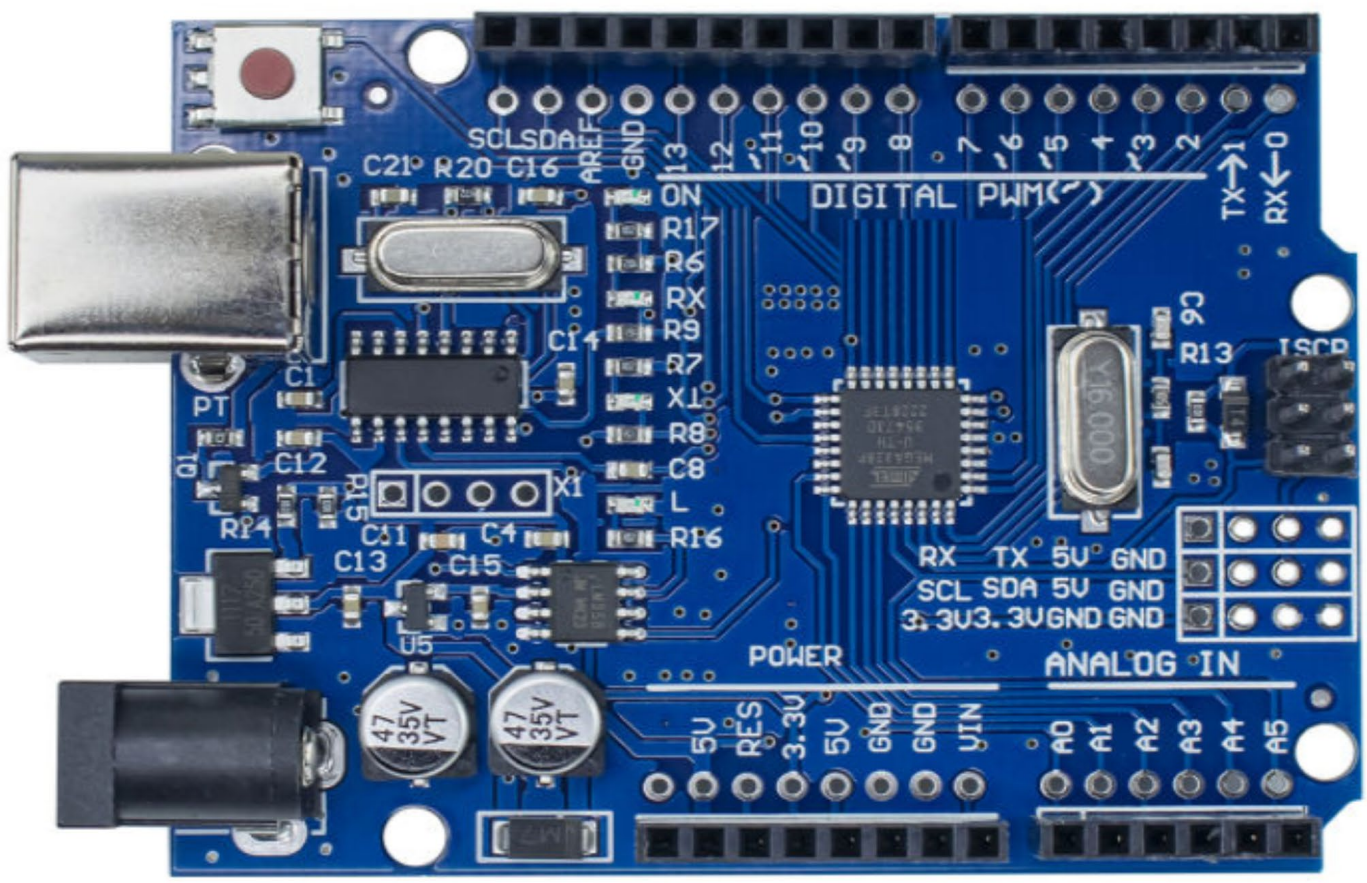
A microcontroller chip^29^.

A basic Li-Fi circuit consists of a light source (laser/LED) Fig. 1, a light collector Fig. 2, and a device to connect and enable them to work (microcontroller) Fig. 3. So, the first step in this research is using a laser as a light source, a solar panel to collect laser light, and an Arduino Uno chip, which is the most valuable and important component that manages the connection between the other components such as data entry and display device. The technical specifications for the Uno chip are shown in Table 3.

**Table 3:** Arduino UNO technical Specs^30^.

Metric	Value
Main processor	ATmega328P 16 MHz
USB-Serial processor	ATmega16U2 16 MHz
Memory	2 KB SRAM, 32 KB FLASH, 1 KB EEPROM
I/O voltage	5 V
DC Current Per I/O Pin	20 mA
Number of pins	13 built-in 14 digital I/O 6 analog inputs

**Fig. 4:**
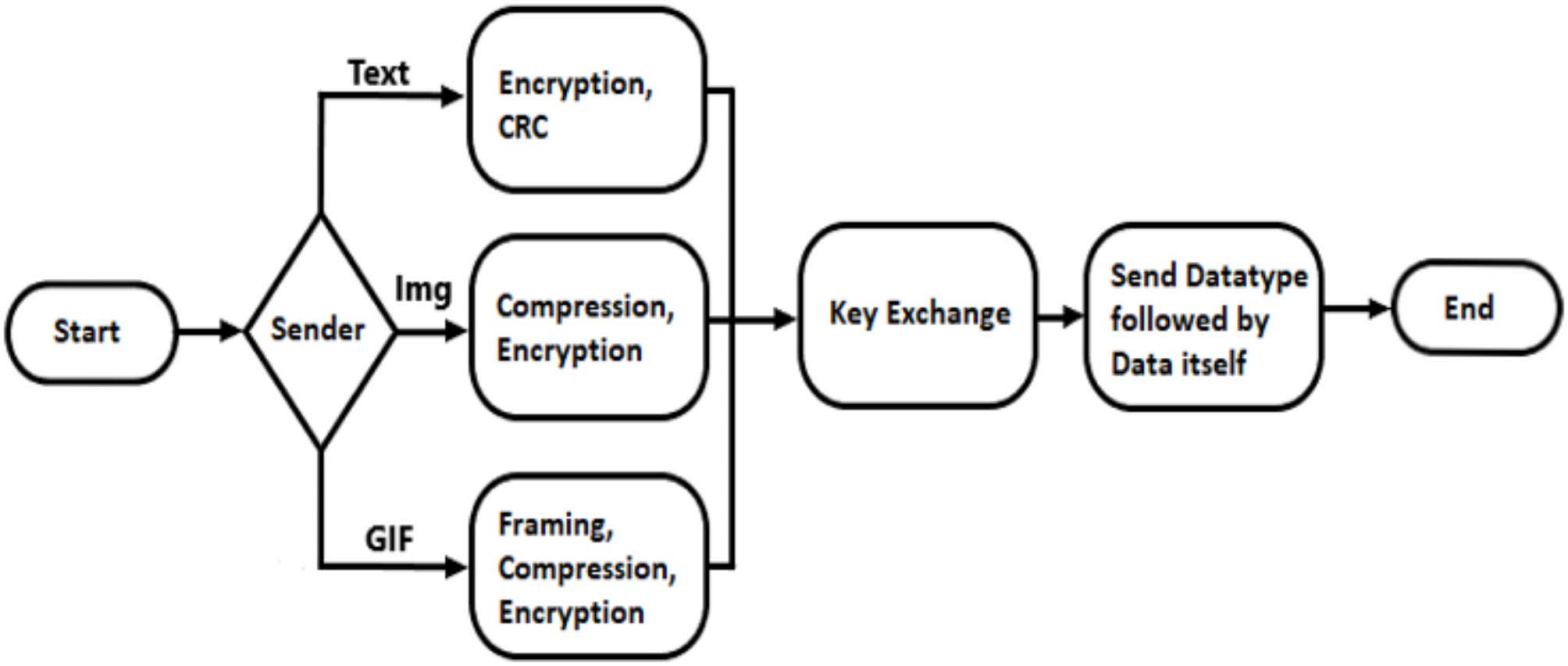
Flowchart of the sender side.

**Fig. 5:**
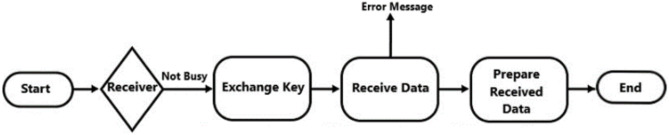
Flowchart of the receiver side.

The proposed system starts with two data entry devices, the sender is connected to the laser, and the receiver is connected to the solar panel. The sender enters the message wanted to be sent, this message will be prepared according to its data format and then converted to binary format, the binary data are considered as the laser modes, so **1** means the laser will be **ON** otherwise the laser will remain **OFF**. An intermediate step is exchanging the security key, which will be used for decryption steps. At the receiver side, the receiver will check if the solar panel read value higher than the predefined threshold, which means that there is a light source trying to send a bit. The collected values are then prepared by inversing all the sender’s preparation steps according to its data format. The process is illustrated in Figs. 4 and 5.

### Reliability

A reliable communication protocol informs the sender if the data has been successfully delivered to the intended recipients and guarantees that all transmitted data is complete and can be correctly reassembled by the receiver in the proper sequence.

The first method used was just an echo message, which was sent by the receiver as an acknowledge message to the sender, this only declares that the message has been received but the issue is it doesn’t guarantee that the data was received correctly. So, this method must have been enhanced. The Li-fi system achieves this reliability by using the CRC algorithm which is a method to detect accidental changes/errors in the communication channel between the transmitter and the receiver.

At the Sender Side, the CRC code is calculated by applying long division on the data and sending the result of the calculations at the end of the transmitted data. On the other hand, the Receiver gets the data and the CRC code, and applies the long division operation. If the reminder is zero, the received data is correct. If the result is not equal to zero, the message is not received correctly. The receiver then, must inform the sender to retransmit the data again by sending an error message to the sender. The sender resends the message till the result of the long division is equal to zero.

### Security

As it is known, Li-Fi technology does not spread like Wi-Fi technology, it spreads along in a straight line, this makes it safer than Wi-Fi, but it remains completely unsecured, so just the presence of the hacker on the communication line (i.e. man in the middle) can recognize the transmitted data. Thus, a layer of protection needs to be added to any Li-Fi system to make sure that no unintended one can know the content of the connection. Therefore, it is preferred to encrypt any data during the communication process to make it secure.

As a start, the ability of the system was checked to choose the most appropriate encryption technique, starting with a very simple encryption algorithm, such as the affine cipher, with a fixed key for both the sender and the receiver using this key to encrypt the data before converting it into binary form. We implemented the technique found in^31^.

Since the affine cipher key was fixed, the method was not secure and needed to be enhanced, which led to trying another advanced encryption algorithm called Rivest–Shamir–Adleman (RSA), which is commonly used in wireless communications. The RSA belongs to asymmetric cryptography, so there is a variable encryption key for each connection. However, it is complex, significantly slow, and vulnerable to side-channel attacks according to^32^.

Another advanced encryption algorithm is the Advanced Encryption Standard (AES). It is the most secure encryption algorithm and the only one approved by the National Security Agency (NSA)^33^. The AES is a modern block cipher that provides excellent long-term security against brute-force attacks, it provides strong diffusion and confusion, and it is efficient in software and hardware. We used a 128-bit version with 10 rounds of keys. For the key exchange process, we have used the Diffie-Hellman algorithm, which is a specified algorithm for exchanging public/private pairs of keys between sender and receiver^34^.

### Hardware

Since we use Li-Fi in our system, the characteristics of Li-Fi were taken into consideration. Li-Fi employs visible light as an optical carrier for data transfer and illumination in the electromagnetic band between 400 THz and 800 THz. When using wireless media, it transmits information via quick light pulses that are invisible to the human eye with speeds up to 100 Gbps. As it is known, any communication system consists of two sides, the sender side, and the receiver side, so the main components of the system contain the following:

- **Laser diode:** Which, acts as a transmission source.
- **Solar cell panel:** Which, acts as the receiving element.

In this communication system, full-duplex mode was used, which means that the sender and the receiver have the same components and any one of them can become a sender or a receiver at any time.

**Fig. 6:**
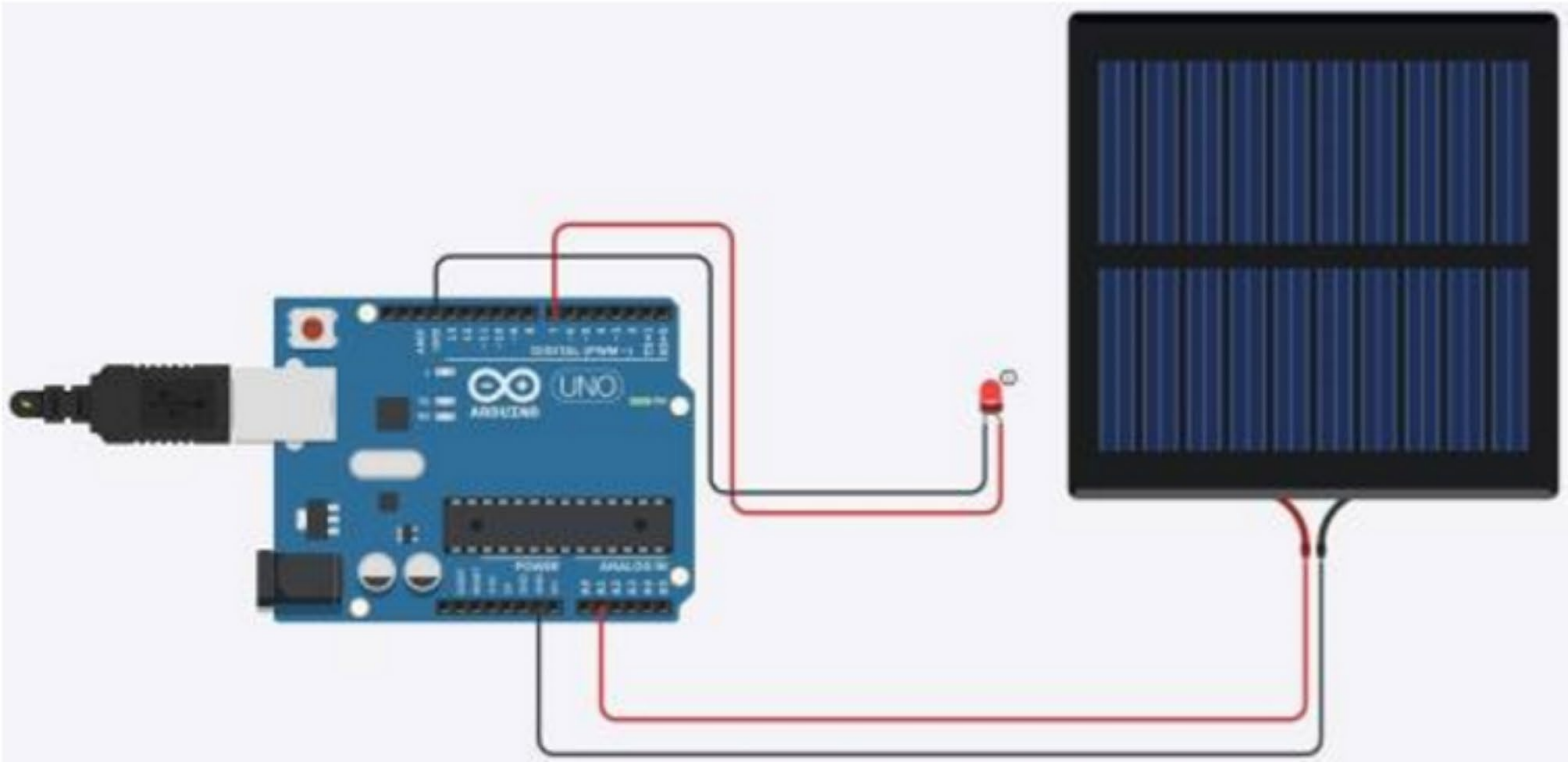
Simulation of the system circuit.

**Fig. 7:**
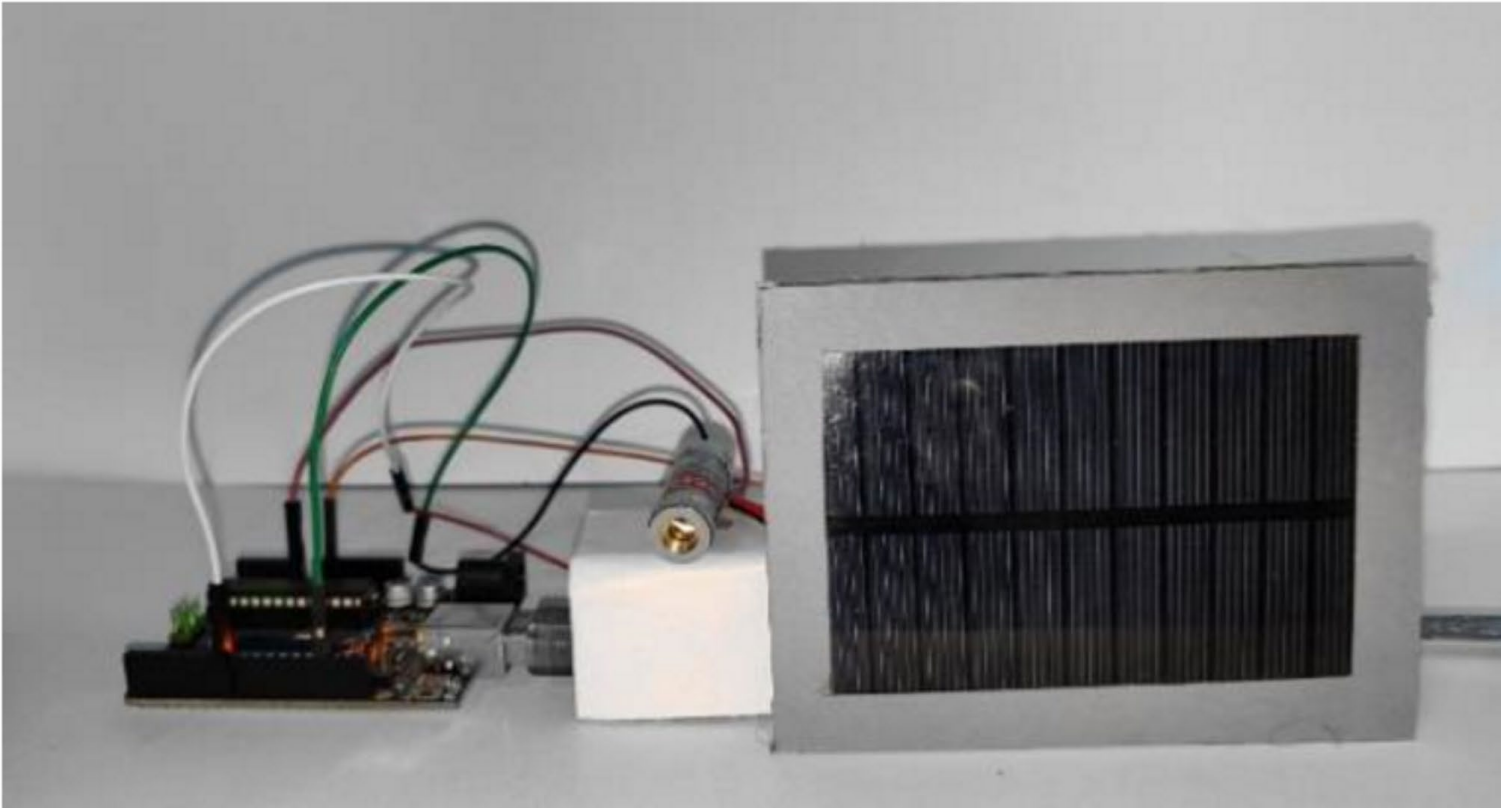
The system circuit.

As it is known, the data is a combination of **1s** and **0s**. The laser forms digital strings with various combinations of **1s** and **0s** by being turned **ON** and **OFF**. By adjusting the laser’s flicker rate, data is encoded within the light, generating a continuous data stream. Through this modulation of the light, the laser effectively functions as a high-speed transmitter. The transmitter or the receiver system consists of four primary components an Arduino chip, laser/LED, solar cell panel, and wires. As shown in Figs. 6 and 7.

### System experiments and results

**Fig. 8:**
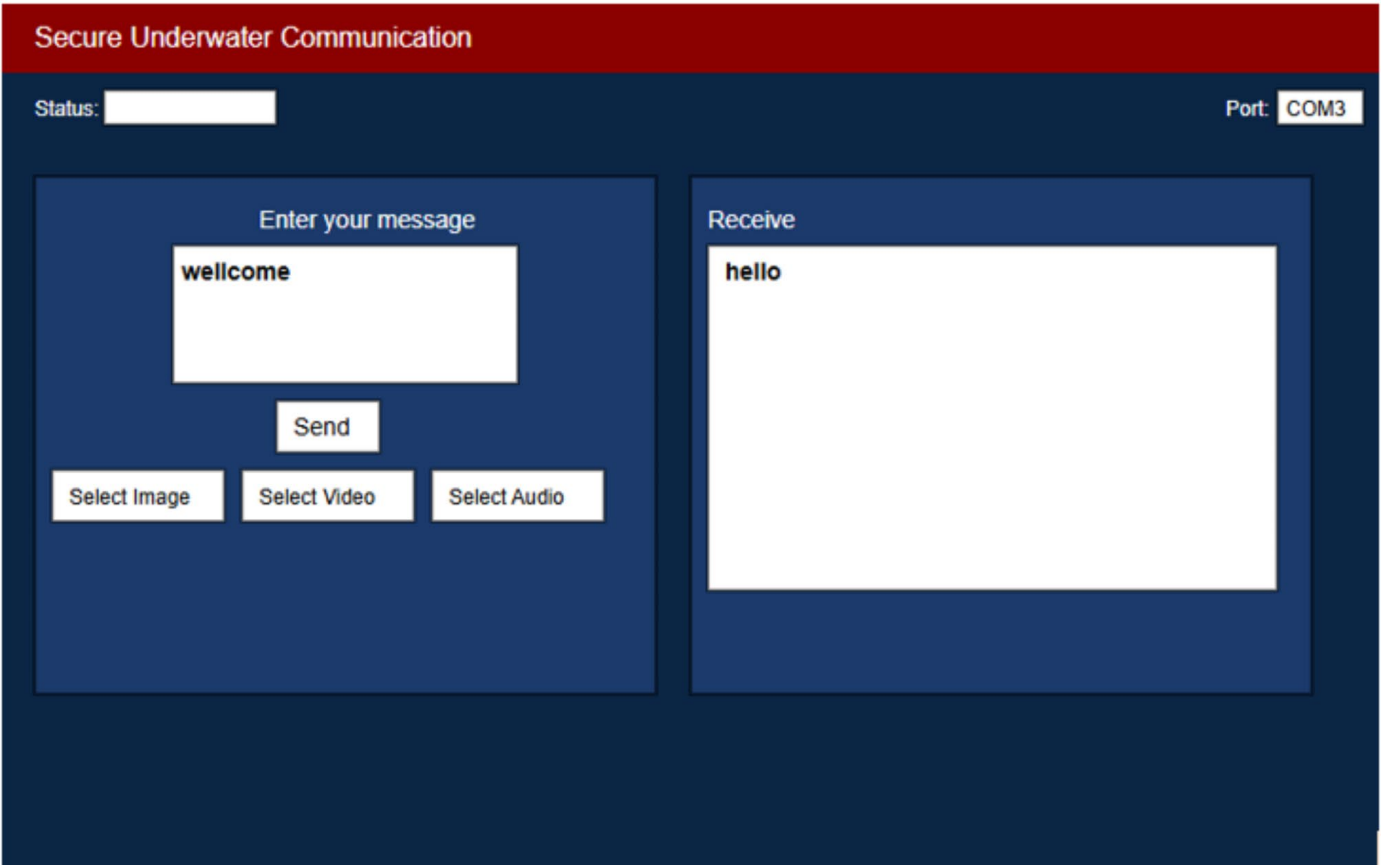
The system’s GUI.

The system was tested in a fish tank with fresh, clear, and wave-free water to simulate an underwater environment. We successfully transmitted and received various data formats, including text, images, and GIF files. Users can easily select the desired data format from the implemented GUI Fig. 8 before initiating communication. Additionally, several features were tested to ensure both data reliability and protection.

The experiment flows as follows:

- The sender inputs the message, and the system encrypts it using the AES encryption algorithm before transmission, specifically a 128-bit AES version with 10 rounds of key generation was employed.
- The Diffie-Hellman key exchange algorithm was used to securely exchange cryptographic keys over a public channel.
- Upon receiving the message, the recipient decrypts it.
- To ensure reliability, the system uses the CRC method to verify that the message is transmitted accurately and without errors.

The sending and receiving process in the proposed system is a straightforward process consisting of two steps:

- **On the Sender Side:** The sender selects the data format and inputs the data. The sending system then communicates with the receiving system to exchange encryption keys. The data is encrypted using AES, followed by the CRC process to ensure accuracy. The data is then sent through the public channel to the receiving system.
- **On the Receiver Side:** Upon receiving the data, the system performs a CRC check to verify its integrity. If errors are detected, an error message is sent back to the sender, requesting the data be resent. If the data is correct, the system proceeds to decryption and displays the message.

The effectiveness of the proposed system is clearly demonstrated by its ability to successfully transmit and receive text, audio, images, and GIF files in full-duplex mode. Table 4 provides a comparative analysis of the proposed Li-Fi-based underwater communication system against existing works. It highlights key aspects such as communication mode, supported data types, system range, security techniques, and reliability mechanisms that have been used in the system.

**Table 4:** Comparison of the proposed system with existing works in Li-Fi-based underwater communication.

References	Tested underwater	Communication mode	Supported data types	Range (meter)	Use security techniques	Use reliability mechanisms
^12^	No	–	–	–	No	No
^13^	Yes	–	Text, audio, and video	–	No	No
^14^	Yes	–	Sensor reads	–	No	No
^15^	Yes	–	Sensor reads	–	No	No
^16^	Yes	Half–duplex	–	Short Range	No	No
^17^	Yes	–	–	–	No	No
^18^	Yes	–	–	–	No	No
^19^	Yes	–	–	–	No	No
^20^	Yes	–	Audio, Video, and text	Short Range	No	No
^21^	Yes	–	–	100 Meters	No	No
^22^	Yes	–	Audio	–	No	No
^23^	Yes	Full–duplex	Sensor reads	–	No	No
^24^	Yes	–	Text, audio, and images	–	No	No
^25^	No	–	Text and audio	–	No	No
^26^	Yes	–	–	–	No	No
Proposed system	Yes	Full–duplex	Text, audio, image, and video	Short Range	Yes	Yes

– Not mentioned.

The **Tested Underwater** column in the table is marked **Yes** for any study that explicitly mentions underwater communication. Additionally, since most studies do not specify the communication mode, we assume it to be simplex by default. Similarly, studies that do not clarify the type of transmitted data are presumed to focus on sensor data. Furthermore, as the majority of studies do not mention the transmission range, it is reasonable to indicate that their range is limited to a short range.

The comparison table highlights the lack of comprehensive studies that propose a fully tested and functional Li-Fi-based underwater communication system. Many critical parameters are missing in almost all the reviewed studies, underscoring the need for further research in this field.

## Proposed system use cases

The proposed Li-Fi-based system is designed to support a diverse range of underwater applications, addressing critical challenges in data transmission reliability, and security. Its adaptability makes it suitable for various domains, including marine research, environmental monitoring, defense, and underwater robotics. The system can facilitate real-time data exchange in challenging aquatic environments. Additionally, the integration of reliability and security mechanisms ensures robust performance, making it a viable solution for mission-critical underwater operations.

### Underwater communications

The proposed system can be used to transmit data at high speeds between underwater devices such as vehicles, robots, and sensors. Using specialized light-emitting devices and receivers, data can be transmitted optically through water, offering faster, secure, and more reliable communication than traditional methods.

### Underwater monitoring

The system can enable real-time monitoring of underwater environments. Sensors equipped with Li-Fi capabilities can gather data on water temperature, pressure, salinity, and other environmental factors, and transmit this information to a central hub for analysis and decision-making.

### Underwater exploration

The system can support underwater research and exploration. Remotely operated vehicles (ROVs) and autonomous underwater vehicles (AUVs) can use Li-Fi communication to transmit high-definition images, videos, sensor data, and even the control information between several AUVs^35^ in real-time. This technology aids scientists in studying marine life, mapping ocean floors, and exploring underwater caves or shipwrecks.

### Underwater surveillance

The system can enhance underwater surveillance by enabling high-bandwidth communication between underwater cameras and monitoring stations. This would allow faster and more reliable transmission of video feeds, making it ideal for underwater applications.

### Underwater internet connectivity

The proposed system can provide internet connectivity to underwater habitats or structures, such as underwater data centers, offshore oil rigs, and research stations. By establishing a Li-Fi network, data can be efficiently transferred between these installations and the surface, enabling seamless communication and data exchange.

### Underwater archaeology

Li-Fi technology can assist archaeologists in conducting 3D surveys and documenting fragile underwater historical sites with minimal disruption, offering a more precise and non-invasive approach to underwater archaeology.

### Scuba diving communication

For enhanced safety and connectivity, scuba divers can use the proposed system through wearable devices like wrist modules or head-up displays, allowing them to communicate and share data, while underwater.

## Conclusion and future work

Li-Fi technology presents a transformative solution for underwater communication, offering a high-speed, secure, and interference-free alternative to traditional methods. In this study, we proposed a reliable and secure Li-Fi-based underwater communication system, incorporating well-established security mechanisms such as AES encryption and reliability techniques like CRC error detection. Our system successfully demonstrated the transmission of various data formats, including text, images, audio, and GIFs, in a full-duplex mode.

Beyond underwater applications, the proposed system has potential use cases in diverse environments such as hospitals, offices, retail stores, and smart homes, where strong and continuous light sources are readily available for data transmission. This versatility underscores the broader impact and adaptability of Li-Fi technology.

For future work, we plan to enhance the system’s data transmission speed and extend its operational range by integrating advanced modulation techniques and optimizing hardware components. Additionally, we aim to implement and test the system in real-world underwater conditions to evaluate its performance under varying water parameters such as muddiness and saltness. A key focus will be the development of an adaptive communication protocol that dynamically adjusts to environmental factors, improving data reliability and system robustness.

## Data Availability

No specific data were used in this study. The system’s testing involved using a single image, audio file, and GIF obtained from publicly available online sources. These files were used to test the system’s ability to send and receive different data formats. Some media samples can be found in^36^.

## References

[1] Dong, C. et al. The model and characteristics of polarized light transmission applicable to polydispersity particle underwater environment. *Opt. Lasers Eng.* **182**, 108449 (2024).

[2] Ganesan Devi, Jayanthi N., Rahul S., Karthick M., Srinivasan Gokul, Anand M. A critical review on Li-Fi technology and its future applications. *AIP Conference Proceedings*. (2023).

[3] Alfattani, S. Review of LiFi technology and its future applications. *J. Opt. Commu.* **42**, 121 (2018).

[4] Lee, C., Islim, M. S., Videv, S., Sparks, A., Shah, B., et al. Advanced LiFi technology: Laser light. Conference: *Light-Emitting Devices, Materials, and Applications XXIV*. **38**. (2020).

[5] Satvik, G., Roy, D., Bose, S., Dixit, V. & Kumar, A. Illuminating the future: A comprehensive review of visible light communication applications. *Opt. Laser Technol.* **177**, 111182 (2024).

[6] Haas, H. A light-connected world. *Phys. World* **29**, 30–34 (2016).

[7] Shetty, A. A Comparative study and analysis on Li-Fi and Wi-Fi. *Int. J. Comput. Appl.* **150**, 6 (2016).

[8] Wikipedia: Radio spectrum. https://en.wikipedia.org/wiki/Radio_spectrum

[9] Wikipedia: Light. https://en.wikipedia.org/wiki/Light

[10] Nasir, S., Çelik, A., Al-Naffouri, T. & Alouini, M. S. Underwater optical wireless communications, networking, and localization: A Survey. *Ad Hoc Netw.* **94**, 101935 (2019).

[11] Wikipedia: Sonar. https://en.wikipedia.org/wiki/Sonar

[12] Balaka, B., Nakhale, A. & Sinha, A. Lighting up data: The future of wireless data transfer with Li-Fi technology. *Telecommun. Radio Eng.* **83**, 59–83 (2024).

[13] Thilagavathy, P., Kavitha, P. R., Therasa, S., Fazliya, R., Evangline, S. Diksha Sen. Audio, video, image, text data transmission in undersea using light fidelity. In: *IEEE International Conference for Women in Innovation, Technology & Entrepreneurship (ICWITE), Bangalore, India*. 258–264. (2024).

[14] Rashid, A., Naimul, I., Sadia, M. Abdullah, Anik. Li-Fi technology enabled unmanned underwater vehicle: Combined cybernetics application and implementation of underwater long-range communication and maritime safety*.* In:* 2nd International Conference on Mechanical Engineering and Applied Sciences(ICMEAS)*. (2022).

[15] Karthikeyan, K. K., Praveen, N., Rajesh, N., Ram, C. Sanjay. G. Submarine communication for monitoring diver’s health using Li-Fi. In:* 7th International Conference on Computing Methodologies and Communication (ICCMC)*, *Erode, India*. 954–961. (2023).

[16] Lata, Y. R., Gusain, R., Shekhar, S., Vidyarthi, A., Prakash, R. Gowri. R. Development of a LiFi system for underwater communications. In: *9th International Conference on Signal Processing and Communication (ICSC), India*. 99–102. (2023).

[17] Thorat, S. Deep water communication using Light Fidelity (Li-Fi). *Int. J. Sci. Res. Public.* **12**, 11 (2022).

[18] Krishnamoorthy, N., Ajit, G., Rajalakshmi, G., Marshiana, D. Delsy Kennedy. An automated underwater wireless communication system using Li-Fi with IOT support and GPS positioning. *Journal of Physics: Conference Series*. 1770. (2021).

[19] Muhammad, A., Qiao, G. Muzzammil, M. Design and Analysis of Li-fi Underwater Wireless Communication System. In: Conference: *OES China Ocean Acoustics (COA).*1100–1103. (2021).

[20] Imran, M., Bai, Y. & Javid, M. Data (Audio, Video, and Image) transmission through visible light communication: LiFi technology. *North Am. Acad. Res.* **4**, 245–256 (2021).

[21] Rajan, S., Srivastava, N. C, V. M. B.S, A. Karadi, V. M R, D. S. Development of underwater communication system using Li-Fi technology. In: *5th International Conference on Circuits, Control, Communication and Computing (I4C), Bangalore, India.* 509–514. (2024).

[22] M. A. J. A, A. B, A. B, B. M, C. RL, Underwater communication using Li-Fi. *3rd International Conference on Signal Processing and Communication (ICPSC), Coimbatore, India*. 485–489. (2021).

[23] Mahalakshmi, S. et al. Seamless subaquatic connectivity: Harnessing Li-Fi for Underwater Communication. V. M R, D. S. *International Conference on Electrical Electronics and Computing Technologies (ICEECT), Greater Noida, India*. 1–6. (2024).

[24] Mahakal, J., Mahajan, J., Mujage, V., Patil, G. & Gaikwad, V. Underwater wireless communication using Li-Fi technology. *Indian J. Techn. Edu.* **1**, 74–80 (2021).

[25] Shirisha, J., Vineela, M., Sri Krishna, P., Mahammad, M. D. & Datta, K. B. LiFi based voice audio communication. *Int. J. Math. Model. Simulation Appl.* **16**, 88–96 (2024).

[26] Seetharaman, R., Tharun, M., Sreeja Mole, S.S. Anandan, K. Application of Li-Fi technique in underwater communication. In: *Materials Today: Proceedings*. **51**, 2249–2253. (2022).

[27] UGE Electronics: KY-008 5mw Laser Transmitter Module Arduino Compatible. https://uge-one.com/product/ky-008-5mw-laser-transmitter-module-arduino-compatible/

[28] Mobile Solar: Solar Panel 120Wp Light & Flat, Flexible. https://mobile-solar.ch/solar-flexible/solar-panel-120wp-light-flat-flexible.html?language=en

[29] https://www.lazada.com.ph/products/r3-board-for-arduino-uno-r3-ch340mega328p-chip-16mhz-for-arduino-r3-development-board-with-cable-i4210166096.html

[30] Arduino DOCS: UNO R3. https://docs.arduino.cc/hardware/uno-rev3/#tech-specs

[31] The Affine Cipher. https://medium.com/infosec-adventures/the-affine-cipher-de3998d363bc (2018).

[32] RSA Algorithm in Cryptography. https://www.geeksforgeeks.org/rsa-algorithm-cryptography/ last updated (2024)

[33] AES: How the most advanced encryption actually works. https://medium.com/codex/aes-how-the-most-advanced-encryption-actually-works-b6341c44edb9 (2021)

[34] Alexander s. Gillis. Diffie-Hellman key exchange (exponential key exchange). https://www.techtarget.com/searchsecurity/definition/Diffie-Hellman-key-exchange (2022).

[35] Chen, B., Hu, J., Zhao, Y. & Ghosh, B. K. Finite-time observer based tracking control of uncertain heterogeneous underwater vehicles using adaptive sliding mode approach. *Neurocomputing* **481**, 322–332 (2022).

[36] google drive: https://drive.google.com/drive/folders/18lTuwGggHU8MwHK0-E4S9gDR-euyTc6Y?usp=drive_link

